# Climate-driven increase of natural wetland methane emissions offset by human-induced wetland reduction in China over the past three decades

**DOI:** 10.1038/srep38020

**Published:** 2016-11-28

**Authors:** Qiuan Zhu, Changhui Peng, Jinxun Liu, Hong Jiang, Xiuqin Fang, Huai Chen, Zhenguo Niu, Peng Gong, Guanghui Lin, Meng Wang, Han Wang, Yanzheng Yang, Jie Chang, Ying Ge, Wenhua Xiang, Xiangwen Deng, Jin-Sheng He

**Affiliations:** 1 State Key Laboratory of Soil Erosion and Dryland Farming on the Loess Plateau, Northwest A&F University, Yangling, 712100, China; 2 Department of Biology Science, Institute of Environment Sciences, University of Quebec at Montreal, Montreal, H3C 3P8, Canada; 3 Western Geographic Science Center, US Geological Survey, Menlo Park, CA 94025, USA; 4 International Institute for Earth System Science, Nanjing University, Nanjing, 210093, China; 5 Earth Science and Engineering, Hohai University, Nanjing, 210098, China; 6 Chengdu Institute of Biology, Chinese Academy of Sciences, Chengdu, 610041, China; 7 State Key Laboratory of Remote Sensing Science, Institute of Remote Sensing and Digital Earth, Chinese Academy of Sciences, Beijing, 100101, China; 8 Ministry of Education for Earth System Modeling, Center for Earth System Science, Tsinghua University, Beijing, 100084, China; 9 College of Life Sciences, Zhejiang University, Hangzhou, 310058, China; 10 National Engineering Laboratory for Applied Technology of Forestry&Ecology in South China, Central South University of Forestry and Technology, Changsha, 410004, China; 11 Department of Ecology, Peking University, Beijing, 100871, China

## Abstract

Both anthropogenic activities and climate change can affect the biogeochemical processes of natural wetland methanogenesis. Quantifying possible impacts of changing climate and wetland area on wetland methane (CH_4_) emissions in China is important for improving our knowledge on CH_4_ budgets locally and globally. However, their respective and combined effects are uncertain. We incorporated changes in wetland area derived from remote sensing into a dynamic CH_4_ model to quantify the human and climate change induced contributions to natural wetland CH_4_ emissions in China over the past three decades. Here we found that human-induced wetland loss contributed 34.3% to the CH_4_ emissions reduction (0.92 TgCH_4_), and climate change contributed 20.4% to the CH_4_ emissions increase (0.31 TgCH_4_), suggesting that decreasing CH_4_ emissions due to human-induced wetland reductions has offset the increasing climate-driven CH_4_ emissions. With climate change only, temperature was a dominant controlling factor for wetland CH_4_ emissions in the northeast (high latitude) and Qinghai-Tibet Plateau (high altitude) regions, whereas precipitation had a considerable influence in relative arid north China. The inevitable uncertainties caused by the asynchronous for different regions or periods due to inter-annual or seasonal variations among remote sensing images should be considered in the wetland CH_4_ emissions estimation.

Methane (CH_4_) is the second most important greenhouse gas following carbon dioxide (CO_2_), and has 28 times the global warming potential of CO_2_ over a 100-year time horizon^1^. Small changes in atmospheric CH_4_ concentrations would have significant implications for climate change^2^. The growth rate of atmospheric CH_4_ slowed in the 1990 s and leveled between 1999 and 2006^3^, but renewed growth was observed since 2007^4^,^5^. Quantification of the sources and sinks of atmospheric CH_4_ and a better understanding of the CH_4_ budget are critically important for reducing uncertainties in future climate change projections^3^,^6^. Wetlands are considered to be the largest contributor (more than 75%) to natural CH_4_ emissions, accounting for more than 20% of the global CH_4_ source^7^,^8^,^9^. The variation in wetland CH_4_ emissions has been considered as a dominant factor in explaining the fluctuations in the atmospheric CH_4_ concentrations over the past two decades^4^,^5^,^10^. Thus, it is important to quantify the strength of natural wetland CH_4_ emissions^3^, and the estimations of these emissions at a regional scale are urgently needed^9^,^11^.

Anthropogenic activities are believed to have major effects on wetland ecosystems which are fragile and sensitive to hydrologic shifts and land-use changes^12^. China’s wetlands have suffered severe losses over the past 50 years, mainly due to the reclamation of land for agricultural purposes^13^, which have a large impact on CH_4_ emissions^14^. Recent study suggested that conversion of marshland to cropland in northeast China between 1950 and 2000 resulted in a 28 Tg cumulative reduction in CH_4_ emissions and a reduced greenhouse effect^14^. Seasonal and inter-annual wetland area variations are among the largest uncertainties in the global CH_4_ budget^15^. The estimation accuracy for wetland CH_4_ emissions is limited by poor quantifications of wetland areas and global wetland distributions^2^,^8^,^15^. Recently, remote sensing data have been used to derive global inundated areas and wetland distributions^16^,^17^,^18^ and to investigate the effects of changes wetland area on CH_4_ emissions^19^.

Over the past three decades, a number of studies on CH_4_ emissions from natural wetlands in China have been conducted, but the estimation of wetland CH_4_ emissions through site-based measurement extrapolation is still preliminary due to the coarse data of wetland areas and distributions^20^,^21^,^22^,^23^. The inventory method does not reflect the spatial heterogeneity in regional wetland CH_4_ emissions and the effects of wetland area dynamics. Based on remotely sensed wetland dynamics information (four periods: 1978, 1990, 2000, and 2008)^24^, we used the process-based model of TRIPLEX-GHG with a full description of wetland CH_4_ emission processes^25^,^26^ to quantify the effects of changing climate and wetland area on the regional CH_4_ budget in China.

## Results

### Historical spatiotemporal patterns of wetland CH_4_ emissions in China

**Figure 1:**
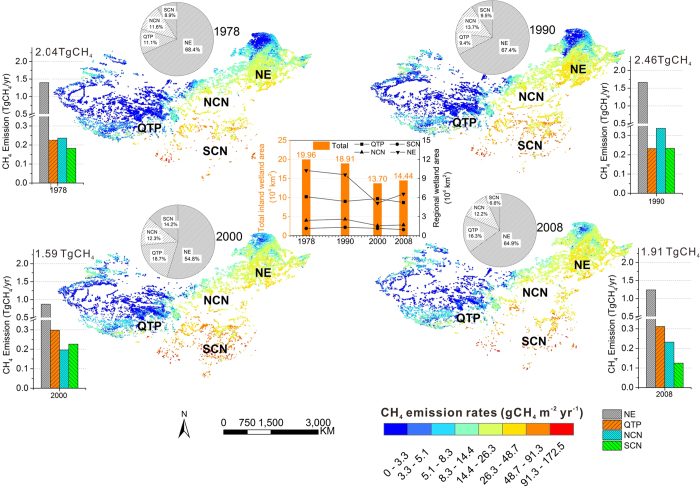
National and regional wetland area dynamics (Bar and symbol line), CH_4_ emissions from wetlands (Bar), contribution percentage of each region to national wetland CH_4_ emissions (Pie), and spatial patterns of wetland CH_4_ emission rates with specific wetland distribution information (maps) of China in four periods (1978, 1990, 2000 and 2008). Regions: Northeast China (NE), Qinghai-Tibet Plateau (QTP), northern China (NCN), and southern China (SCN). The maps were generated with ArcGIS 10.2, http://www.esri.com/.

From 1978 to 2008, China’s inland natural wetland area decreased by 27.6% (Fig. 1). Relative to 1978, the wetland area decreased 5.2% by 1990 and 31.3% by 2000, and increased 5.4% between 2000 and 2008. Nearly 28% of China’s inland wetlands disappeared between 1990 and 2000 (Fig. 1). The Northeast region (NE), with the largest proportion of wetlands in China, lost 46.5% of its wetland between 1990 and 2000 (Fig. 1). The second largest wetland area of China, located in the Qinghai-Tibet Plateau (QTP), was relatively stable between 1978 and 2008 and had approximately an increasing of 8% between 1990 and 2000. The wetland areas in the remaining regions accounted for approximately 18% to 20% of the total over the four periods. The wetlands in NE, QTP, North China (NCN), and South China (SCN) accounted for 45.4%, 36.1%, 11.8% and 6.7%, respectively, of the total in 2008 (Fig. 1).

The wetland CH_4_ emission rate varied significantly across China. The wetlands in SCN showed the highest emission rate (greater than 25 gCH_4_ m^−2^ yr^−1^), followed by NE (15 to 50 gCH_4_ m^−2^ yr^−1^), NCN and QTP (lower than 15 gCH_4_ m^−2^ yr^−1^) (Fig. 1). Over the past three decades, the NE and QTP regions contributed approximately 73% to 81% of the national natural wetland CH_4_ emissions, whereas the NCN and SCN regions made the remaining minor contributions. The total CH_4_ emissions from natural wetlands increased by 21% between 1978 and 1990, but substantially decreased by 35.4% between 1990 and 2000. Then, CH_4_ emissions increased by 20% between 2000 and 2008, reaching 1.91 TgCH_4_ yr^−1^ in 2008 (Fig. 1). The same temporal patterns were detected for the CH_4_ emissions for the NE wetlands with a sharp decrease (47.5%) between 1990 and 2000. The CH_4_ emissions gradually increased in the QTP between 1978 and 2008 and exceeded the emission rates of the NCN and SCN regions in 2000 and 2008 (Fig. 1).

### Effects of wetland area dynamics and climate change on wetland CH_4_ emissions in China

**Figure 2:**
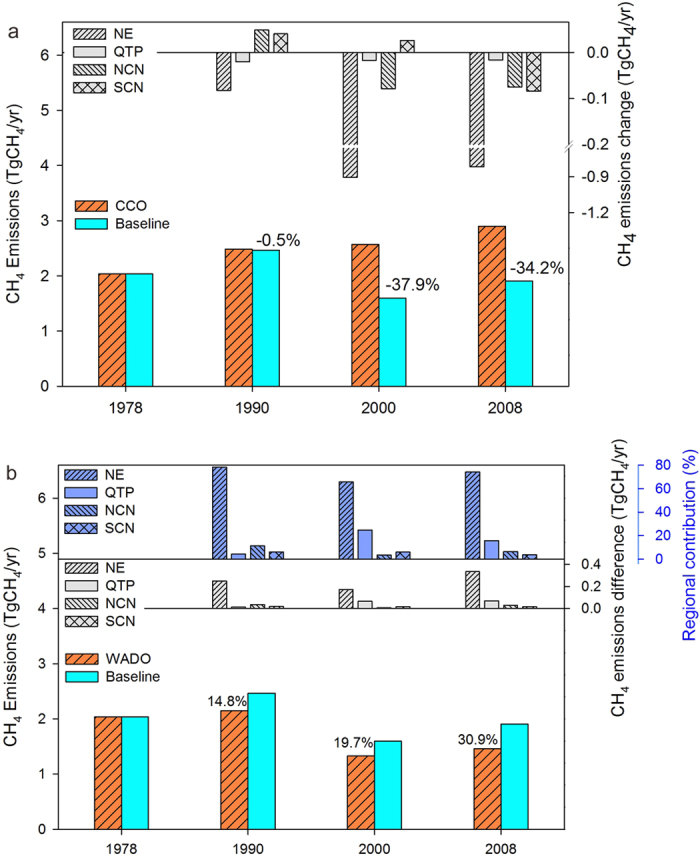
Effects of wetland area dynamics (**a**) and climate change (**b**) on CH_4_ emissions throughout China and in each specific region in four periods (1978, 1990, 2000, and 2008). Numbers above the bars represent the percentage change of wetland CH_4_ emissions between baseline and climate change only simulations (**a**), and between baseline and wetland area dynamics only simulations (**b**). Regions: Northeast China (NE), Qinghai-Tibet Plateau (QTP), northern China (NCN), and southern China (SCN). Simulation scenarios: Baseline, Climate Change Only (CCO), Wetland Area Dynamic Only (WADO). (see Supplementary Table S1).

With the severe reduction in wetland areas, the total wetland CH_4_ emissions decreased significantly, particularly during last decade. The wetland CH_4_ emissions were 37.9% and 34.2% lower in 2000 and 2008, respectively, with the corresponding wetland areas decreased 31.3% and 27.6%, respectively, relative to 1978 (Figs 1 and 2a). At the regional scale, the wetland area change in the NE region made the largest contribution to decreasing CH_4_ emissions. The annual wetland CH_4_ emissions in this region are reduced by approximately 0.91 and 0.81 Tg CH_4_ in 2000 and 2008 compared to 1978, respectively (Fig. 2a). Climate change alone has enhanced the wetland CH_4_ emissions by 14.8%, 19.7% to 30.9% in 1990, 2000, and 2008, respectively (Fig. 2b). Additionally, the enhancements of wetland CH_4_ emissions was greatly contributed by NE region which accounted for 78.3%, 65.8%, and 74.1% of the national total enhancement in 1990, 2000, and 2008, respectively. Climate change had a relatively greater positive effect on CH_4_ emissions from the NE and QTP wetlands than in the other regions, particularly in 2000 and 2008 (Fig. 2b).

**Figure 3:**
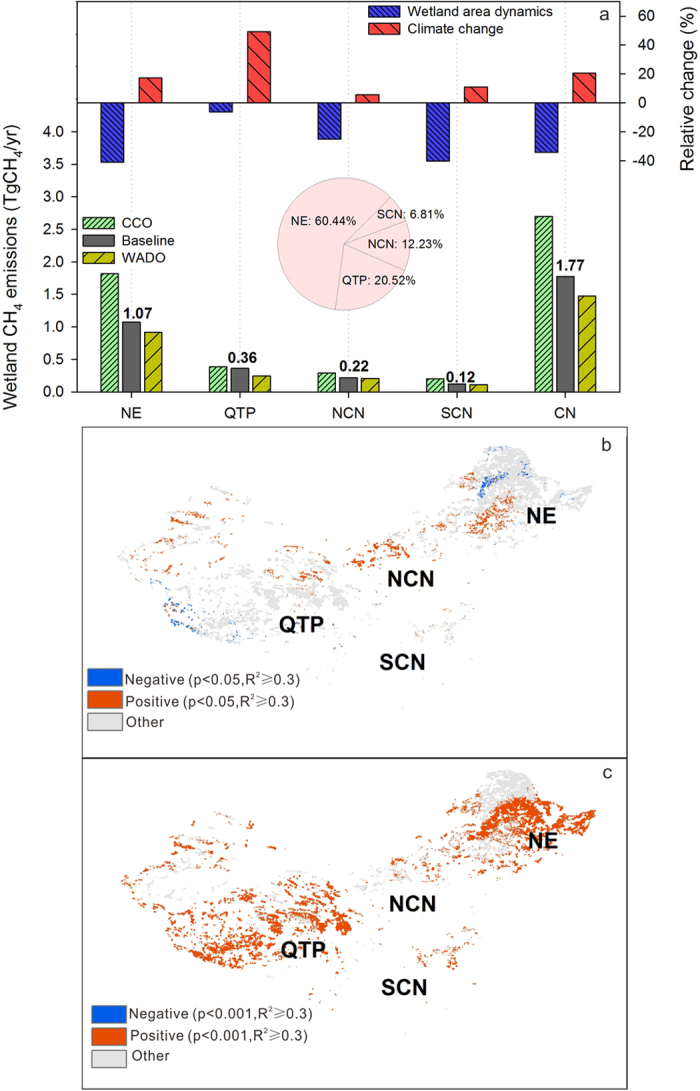
(**a**) Mean annual CH_4_ emissions from wetlands through China (CN) and from each specific region (NE, QTP, NCN and SCN) between 2010 and 2013 and the changes caused by wetland dynamics and climate change. The pie chart represents the percentage contribution of wetland CH_4_ emissions for each specific region to the whole China, and the numbers above the bars represent annual total CH_4_ emissions for each individual region and whole China; **b** and **c**: Correlations between wetland CH_4_ emission rates and precipitation (**b**) and temperature (**c**) for the fixed wetlands (i.e., the areas that remained wetlands from 1978 to 2008). The maps were generated with ArcGIS 10.2, http://www.esri.com/.

The annual wetland CH_4_ emissions (2010–2013) in China were estimated to be 1.77 Tg CH_4_ yr^−1^ based on the wetland area in 2008 (Fig. 3a). The annual wetland CH_4_ emissions in China was reduced by 34.3% (0.92 TgCH_4_) due to wetland loss since 1978 but increased 20.4% (0.31 TgCH_4_) due to climate change (Fig. 3a). At a regional scale, wetlands in the NE and QTP released 1.07 Tg CH_4_ yr^−1^ and 0.36 Tg CH_4_ yr^−1^, or 60.4% and 20.5% of the national wetland CH_4_ emissions, respectively. The NCN and SCN regions in total accounted for approximately 19% of the total wetland CH_4_ emissions in China (Fig. 3a). The reductions in wetland area have greatly reduced CH_4_ emissions particularly in NE, NCN and SCN (by 41.0%, 25.1% and 40.3%, respectively) since 1978. By contrast, CH_4_ emissions were increased due to climate change in all regions. Specifically, climate-induced increases in CH_4_ emissions in the QTP region reached 49.3%. Overall, the negative effects of wetland area reductions are greater than the positive effects of climate change on wetland CH_4_ emissions, except in the QTP (Fig. 3a).

### Relationship between climate (precipitation and temperature) and wetland CH_4_ emissions in China

A further investigation based on a long-term analysis (1951–2013) conducted on the fixed wetlands (i.e., the areas that remained wetlands from 1978 to 2008) presented a straightforward effects of precipitation and temperature on wetland CH_4_ emissions. Wetland CH_4_ emissions was significantly (P < 0.05) and positively correlated with precipitation and was responsible for more than 30% of the variations (R^2^ ≥ 0.3) for some northern areas (Fig. 3b), that indicated precipitation had a considerable influence on wetland CH_4_ emissions in this relative arid north China. However, since temperature was extremely and significantly (P < 0.001) correlated to wetland CH_4_ emissions in most areas (R^2^ ≥ 0.3), particularly in the QTP and NE regions (Fig. 3c), it was a dominant controlling factor for wetland CH_4_ emissions in the NE (high latitude) and QTP (high altitude) regions, which also partially the reason for that wetland CH_4_ emissions in the NE and QTP were more sensitive to climate warming than other regions (see Supplementary Information).

## Discussion

Inter-annual variations and distributions of wetlands are considered important sources of uncertainty in global CH_4_ budget estimations^2^,^15^. Recently, with the remote sensing technology, deriving inter-annual and even seasonal dynamics of inundated areas, and then quantifying the impact of wetland area changes on CH_4_ emissions at specific time scales has become feasible^16^,^17^,^19^. The dramatically total wetland loss in China over the past several decades^18^,^27^,^28^,^29^ was primarily caused by land reclamation for agriculture (accounting for 82%)^13^,^14^. However, wetlands have been recovering since 2000 due to government efforts to create wetland nature reserves, wetland restoration and water pollution control, particularly in NE^13^. Additionally, new wetlands have been temporarily generated due to the retreating of glaciers and thawing of permafrost since 1990^24^,^28^.

As a result of a substantial wetland loss occurred between 1990 and 2000, particularly in the NE region, the wetland CH_4_ emissions in China significantly decreased during this period. The net effects of wetland dynamics were larger than those of climate change in the NE, NCN, and SCN regions and throughout the country. An exception to this is in the QTP, where the CH_4_ emissions from wetlands are mainly controlled by climate change and the impacts of human activities on CH_4_ emissions seem to be much weaker than other above-mentioned regions. The CH_4_ emissions from the wetlands of the QTP gradually increased, even though the wetland area slightly decreased from 1978 to 1990 and from 2000 to 2008. Although the wetland area decreased in the NE and throughout China, the CH_4_ emissions increased between 1978 and 1990. Therefore, the positive effects of climate change on CH_4_ emissions could offset the negative effects of slight wetland losses during this period.

The wetland dynamics of China from 1978 to 2008 derived from remotely sensed data, however, exhibited some uncertainties (see Supplementary Information). For example, the wetland distribution along the Yellow and Yangtze Rivers were not reasonably represented in 2008 and was much smaller compared with the other three years. Subsequently, CH_4_ emissions from the wetlands in the NCN and SCN in 2008 were probably underestimated. Remote sensing images for a specific year across the whole country are difficult to collect, and need to span the time window approximately 3–5 years to complete the jigsaw map^29^. The wetland distributions in different regions or for different periods are not synchronous due to inter-annual or seasonal variations among remote sensing images. The inevitable uncertainties should be considered in the wetland CH_4_ emissions estimation. Using multiple sources of remote sensing data and applying a correlation analysis between wetland change and environment factors will reduce the uncertainty and improve the accuracy of wetland mapping. Since the wetland CH_4_ emissions is sensitive to inter- and intra- annual wetland area variations (see Supplementary Information), the lack of the information of seasonal wetland area dynamics currently in this study could add additional uncertainties in the estimation of wetland CH_4_ emissions. Land surface models integrated with detailed hydrological processes could potentially provide an effective method to estimate seasonal wetland area dynamics^26^. Furthermore, since mapping accuracy of individual maps cannot be used to address the mapping accuracy of changes, systematic accuracy assessment should be conducted for wetlands mapping in China in the future improvement^30^,^31^.

Although the conversion of wetlands to cropland may significantly reduce CH_4_ emissions and may counterbalance the release of CO_2_ and the reduction of soil organic carbon (SOC), further research is needed to balance wetland loss and the emissions of different greenhouse gases (CO_2_, CH_4_ and N_2_O) under climate change^14^. Furthermore, wetlands are important for their ecosystem functions and services (e.g., for species diversity and conservation, biogeochemical cycling, and hydrological management). Since approximately 50 percent of the world’s wetlands have been destroyed, it is urgent and an ongoing challenge for wetlands protection and restoration^13^.

## Methods

### Wetland dynamics retrieving

The spatially and temporally explicit wetland dynamics of China were derived from the remote sensing data of Landsat MSS/TM/ETM+ and CBERS-02B^29^, which is the most updated and improved wetland datasets available in China^27^. According to the classification system of the Ramsar Convention on wetlands, the wetland distribution maps in China were produced for four periods (1978, 1990, 2000, and 2008) with three major types: coastal wetlands (including tide zone, marine marshes, estuarine water, estuarine delta, and lagoons), inland wetlands (river wetlands, inland marshes/swamp, and lakes) and artificial wetlands (reservoirs/ponds, artificial channel, seawater fish farms/salt flats and other wetlands such as landscaping and recreational water bodies), while rice paddies were not considered in the classification system^28^,^29^. Only natural inland wetlands (water bodies of lakes and rivers were excluded) were considered for analyzing area dynamics and wetland CH_4_ emissions in this study. For each period (1978, 1990, 2000, and 2008), a mask map of natural inland wetlands was derived. The methanogenesis module in the model of TRIPLEX-GHG was only called up on the wetland grids (the resolution was 10 km and consistent with other forcing data, e.g. climate data) for each period and then the CH_4_ emissions from natural wetlands of China were estimated.

### Methanogenesis modelling

In this study, we used the TRIPLEX-GHG model, in which a methanogenesis module, including three major CH_4_ emission processes (CH_4_ production, CH_4_ oxidation and CH_4_ transportation) and the water table dynamics, was developed and integrated^25^,^26^. The key factors that constrain wetland CH_4_ emission processes, e.g., soil temperature, soil moisture, soil pH and soil redox potential (Eh), are described in the model in detail. CH_4_ production was calculated as a proportion of the heterotrophic respiration with soil temperature, Eh and pH modification factors. CH_4_ is oxidized by aerobic methanotrophic activities in the unsaturated zone of the soil above the water table under the control of the soil CH_4_ concentration, soil Eh and soil temperature. CH_4_ emission processes, including diffusion, ebullition and plant-mediated transportation, were formulated. The model performed reasonably over the collected measurement sites, which covered a wide geographical range of boreal, temperate and tropical regions^25^.

### Model forcing data

A series of spatially and temporally explicit datasets (including climate, soil, vegetation, and topographic datasets) were developed for model simulations. The daily climate driving data, including precipitation, temperature (maximum, minimum and mean), relative humidity, wind speed, and solar radiation, were obtained from the China Meteorological Data Sharing Service System (http://www.cma.gov.cn/2011qxfw/2011qsjgx/). The original daily station-based meteorological data (756 stations from 1951 to 2013) were interpolated at a national scale with a resolution of 10 km using the program ANUSPLIN with the support of Digital Elevation Model (DEM)^32^,^33^. The 1:1,000,000 China soil dataset was used to generate the initial soil carbon content, the fractions of sand, clay, and silt, and the soil pH for each cell. The 1:4,000,000 China vegetation dataset was used for the vegetation initialization of the model^34^. The topographic data of China were derived from the Shuttle Radar Topography Mission (SRTM) DEM dataset.

### Simulation performance

The model performance was evaluated at site level (based on observed data obtained from both chamber and eddy covariance measurement) and national level (see Supplementary Information). To evaluate and separate the effects of wetland area dynamics and climate change on China’s wetland CH_4_ emissions (between 1978 and 2013), simulations were forced with different composition of historical climate data and remote sensing based wetland distribution data (Supplementary Table S1). For each simulation, a 300-year spin-up procedure (an internal speed-up process which allows the model to run up to 40 times of additional soil carbon cycling during one normal simulation day was integrated) running with multi-year (between 1960 and 2000) averaged historical meteorological data, was set up and allowed the ecosystem carbon pools to reach a relative equilibrium state. In each simulation, the wetland distribution was kept unchanged by using the wetland map of 1978, 1990, 2000, or 2008. For different simulations, only result slices of particular years (1978, 1990, 2000, 2008, and 2010–2013) were extracted for analysis (Supplementary Table S1). Zonal statistics were conducted for four regions: Northeast China (NE), the Qinghai-Tibet Plateau (QTP), North China (NCN), and South China (SCN).

## Additional Information

**How to cite this article**: Zhu, Q. *et al*. Climate-driven increase of natural wetland methane emissions offset by human-induced wetland reduction in China over the past three decades. *Sci. Rep.* **6**, 38020; doi: 10.1038/srep38020 (2016).

**Publisher's note:** Springer Nature remains neutral with regard to jurisdictional claims in published maps and institutional affiliations.

## Supplementary Material

Supplementary Information
