# Supplementary material for: Climate-driven increase of natural wetland methane emissions offset by human-induced wetland reduction in China over the past three decades
Source: Sci Rep. 2016 Nov 28;6:38020. doi: 10.1038/srep38020 (PMC5125101; doi:10.1038/srep38020)
Supplement: Supplementary Information [file srep38020-s1.pdf]

SUPPLEMENTARY INFORMATION

Climate-driven increase of natural wetland methane emission offset by human-induced wetland reduction in China over the past three decades

Qiuan Zhu[1,2*], Changhui Peng[2,1*], Jinxun Liu[3], Hong Jiang[4], Xiuqin Fang[5], Huai Chen[6], Zhenguo Niu[7], Peng Gong[8], Guanghui Lin[8], Meng Wang[1], Han Wang[1], Yanzheng Yang[1], Jie Chang[9], Ying Ge[9], Wenhua Xiang[10], Xiangwen Deng[10] , Jin-Sheng He[11]

[1]State Key Laboratory of Soil Erosion and Dryland Farming on the Loess Plateau, Northwest A&F University, Yangling, 712100, China,

[2]Department of Biology Science, Institute of Environment Sciences, University of Quebec at Montreal, Montreal, H3C 3P8, Canada,

[3]Western Geographic Science Center, US Geological Survey, Menlo Park, CA 94025, USA,

[4]International Institute for Earth System Science, Nanjing University, Nanjing, 210093, China,

[5]Earth Science and Engineering, Hohai University, Nanjing, 210098, China,

[6]Chengdu Institute of Biology, Chinese Academy of Sciences, Chengdu, 610041, China,

[7]State Key Laboratory of Remote Sensing Science, Institute of Remote Sensing and Digital Earth, Chinese Academy of Sciences, Beijing, 100101, China,

[8]Ministry of Education for Earth System Modeling, Center for Earth System Science, Tsinghua University, Beijing, 100084, China,

[9]College of Life Sciences, Zhejiang University, Hangzhou, 310058, China,

[10]National Engineering Laboratory for Applied Technology of Forestry&Ecology in South China, Central South University of Forestry and Technology, Changsha, 410004, China,

[11]Department of Ecology, Peking University, Beijing, 100871, China.

* Corresponding author: Changhui Peng (peng.changhui@uqam.ca); Qiuan Zhu (qiuan.zhu@gmail.com)

1. Simulation performance

To evaluate and separate the effects of wetland area dynamics and climate change on China's wetland CH_4 emissions (between 1978 and 2013), simulations were driven with different composition of historical climate data and remote sensing based wetland distribution data (Supplementary Table S1). The 1:1,000,000 China soil dataset was used to generate the initial soil carbon content, the fractions of sand, clay, and silt, and the soil pH for each cell. The 1:4,000,000 China vegetation dataset was used for the vegetation initialization of the model. CO_2 concentrations during the simulation period were composed of two parts. Historical observed CO_2 concentrations derived from in situ air measurements at Mauna Loa Observatory, Hawaii[1] were used for the period 1958–2013. CO_2 concentrations before 1958 were obtained from the IS92a Global CO_2 Concentration Yearly Dataset[2]. For wetland CH_4 emission simulations, a new wetland plant function type (PFT) was added in the model (Supplementary Table S2) and most of the PFT phenological and physiological parameters were adopted from the C3 grass PFT in the original model[3]. The definition of inundation stress effects on gross primary productivity (GPP) of the added PFT in wetlands followed the assumption made by Wania et al. (2009)[4], that sphagnum and C3 graminoids photosynthesis will increase or decrease when water table rises or drops. The wetland PFT would be kept fixed over wetland regions during simulation periods.

For each simulation, a 300-year spin-up procedure, running with multi-year (between 1960 and 2000) averaged historical meteorological data, was set up and allowed the ecosystem carbon pools to reach a relative equilibrium state. For reaching soil carbon equilibrium, the model has an internal speed-up process during the soil spin-up period. It allows the model to run up to 40 times of additional soil carbon cycling during one normal simulation day, which means a 300-year soil carbon spin-up has

about 12,000 years of normal soil carbon cycling. The simulations were split into 5 periods: the spin-up period, 1951-1978, 1979-1990, 1991-2000, and 2001-2013 (Supplementary Table S1). The simulations in spin-up period were forced with multi-year averaged climate data and different wetland distributions. Simulations No. 2 and 3 were ended in 1990. Simulations No. 4 and 5 were ended in 2000 (Supplementary Table S1). In each simulation, the wetland distribution was kept unchanged by using the wetland map of 1978, 1990, 2000, or 2008. For different simulations, only result slices of particular years (1978, 1990, 2000, 2008, and 2010-2013) were extracted for analysis (Supplementary Table S1). The baseline results were extracted from simulations No.1, No.2, No.4, No.6, and No.6 for year of 1978, 1990, 2000, 2008, and average of 2010-2013, respectively (Supplementary Table S1).

Supplementary Table S1. List of simulations performed (The simulations selected as baseline for comparisons were checked by tick mark)

		Simulations	Spin-up (300yr)	1951-1978	1979-1990	1991-2000	2001-2013	Results slice extracted	Baseline composition
Effects of climate change	No. 1	Climate<br>Wetland distribution	Multi-year (1960-2000) averaged<br>1978	Daily observed data<br>1978	Daily observed data<br>1978			1978, 1990, 2000, 2008, mean (2010-2013)	√ (1978)
	No. 2	Climate<br>Wetland distribution	Multi-year (1960-2000) averaged<br>1990	Daily observed data<br>1990	Daily observed data<br>1990			1990	√ (1990)
	No. 3	Climate<br>Wetland distribution	Multi-year (1960-2000) averaged<br>1990	Daily observed data<br>1990	Climate data of 1978<br>1990			1990	
	No. 4	Climate<br>Wetland distribution	Multi-year (1960-2000) averaged<br>2000	Daily observed data<br>2000	Daily observed data<br>2000			2000	√ (2000)
	No. 5	Climate<br>Wetland distribution	Multi-year (1960-2000) averaged<br>2000	Daily observed data<br>2000	Climate data of 1978<br>2000			2000	
	No. 6	Climate<br>Wetland distribution	Multi-year (1960-2000) averaged<br>2008	Daily observed data<br>2008	Daily observed data<br>2008			2008, mean (2010-2013)	√ (2008, mean (2010-2013))
	No. 7	Climate<br>Wetland distribution	Multi-year (1960-2000) averaged<br>2008	Daily observed data<br>2008	Climate data of 1978<br>2008			2008, mean (2010-2013)	
Effects of wetland dynamic	No. 1	Climate<br>Wetland distribution	Multi-year (1960-2000) averaged<br>1978	Daily observed data<br>1978	Daily observed data<br>1978			1978, 1990, 2000, 2008, mean (2010-2013)	
	No. 2	Climate<br>Wetland distribution	Multi-year (1960-2000) averaged<br>1990	Daily observed data<br>1990	Daily observed data<br>1990			1990	
	No. 4	Climate<br>Wetland distribution	Multi-year (1960-2000) averaged<br>2000	Daily observed data<br>2000	Daily observed data<br>2000			2000	
	No. 6	Climate<br>Wetland distribution	Multi-year (1960-2000) averaged<br>2008	Daily observed data<br>2008	Daily observed data<br>2008			2008, mean (2010-2013)	

Table S2 Plant functional types (PFTs) included in the model

No.	PFT
1	Tropical broadleaf evergreen trees
2	Tropical broadleaf drought-deciduous trees
3	Warm-temperate broadleaf evergreen trees
4	Temperate conifer evergreen trees
5	Temperate broadleaf cold-deciduous trees
6	Boreal conifer evergreen trees
7	Boreal broadleaf cold-deciduous trees
8	Boreal conifer cold-deciduous trees
9	Evergreen shrubs
10	Cold-deciduous shrubs
11	C4 grasses
12	C3 grasses
13	Wetland vegetation

2. Model performance evaluation and testing

2.1 Model evaluation at site level

The wetland CH_4 emission modeling performance of TRIPLEX-GHG was evaluated using global field measurements from a previous study[3,5]. Observed data, which were collected from more than 10 studies across China, are used to evaluate the model. The results indicated that the TRIPLEX-GHG model successfully captured the mean and variation in the CH_4 emissions of all available observations in China[3] (Supplementary Fig. S1).

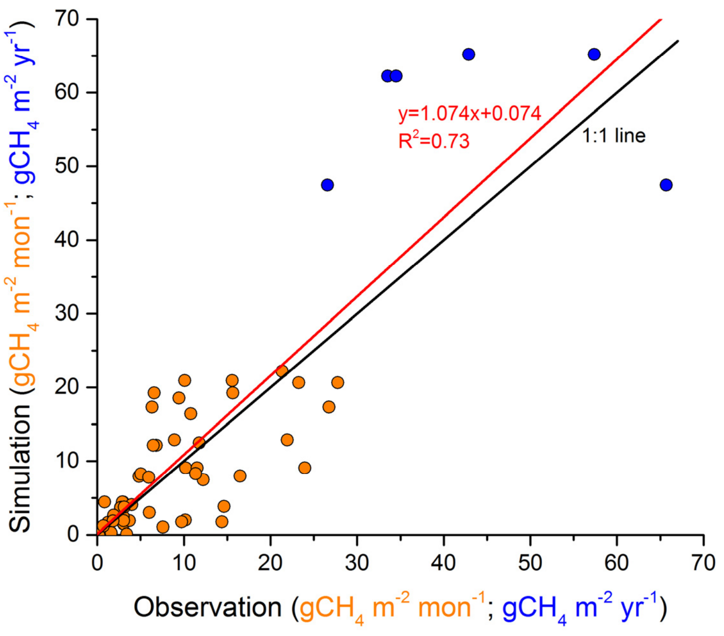

Supplementary Figure S1. Comparison of wetland CH_4 emissions in China between model simulation in this study and site observation in collected literature (Data synthesized from Fig. 4a-4d in the study of Zhu et al.[3])

Three sites in China with specific daily wetland CH_4 emission observations were selected to conduct an additional model performance evaluation at site level. Indices including root mean square error (RMSE), correlation coefficient (r) and index of agreement (D) were used to evaluate the model

performance.

Two sites have static chamber measurement of wetland CH_4 emission: one site is located in the Sanjiang Plain (47°35'N, 133°31'E), which is the largest and most concentrated marsh wetland region in China, and the other site is located in the Zoige Plateau (32°47'N, 102°32'E'), which is the largest peatland region in Qinghai-Tibetan Plateau (QTP) in China. Data was obtained from study of Li et al.[6] and available from 2003 to 2005 for the site of Sanjiang Plain and in 2001 for the site of Zoige Plateau (Supplementary Fig. S2). Simulated seasonal and annual variation agreed well with that of the observation, while it is difficult for the model to capture several peak values during the growing season.

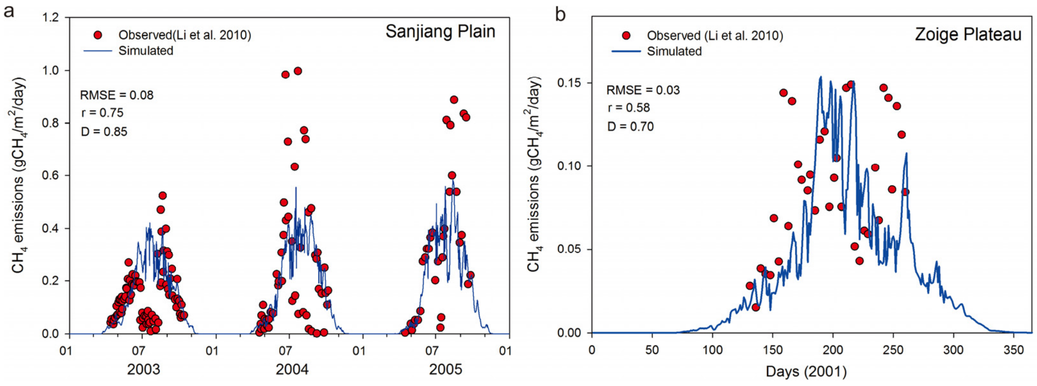

Supplementary Figure S2. Comparison of daily wetland CH_4 emission of two sites located at: a. Sanjiang Plain; b. Zoige Plateau of China

The third site has eddy covariance measurement of wetland CH_4 emission located in Haibei alpine wetland (37°35'N, 101°20'E'), also in Qinghai-Tibetan Plateau. The data was obtained form study of Song et al.[7] and available from 2012 to 2013 (Supplementary Fig. S3). The model captured the seasonal variations of CH_4 emission well with agreement index of 0.87 and 0.91 for daily and monthly

comparison, respectively. However, a rapid increase of CH_4 emission detected in eddy covariance measurement during soil thawing period[7] was not reflected well by the model. In the simulations, wetland CH_4 emission had responses during thawing periods but the responses were not significant as found in the observation. It should be noted that the uncertainties in the gap-filled eddy covariance observation need to be considered in the comparison, particularly in the growing season (gray bar represented the gaps of eddy covariance measurement, Supplementary Fig. S3a). The simulated CH_4 emission was lower than that of observed during non-growing season, particularly in winter (Supplementary Fig. S3a, b), which could be the major reason for that the simulated annual emission was lower than the observation (Supplementary Fig. S3c).

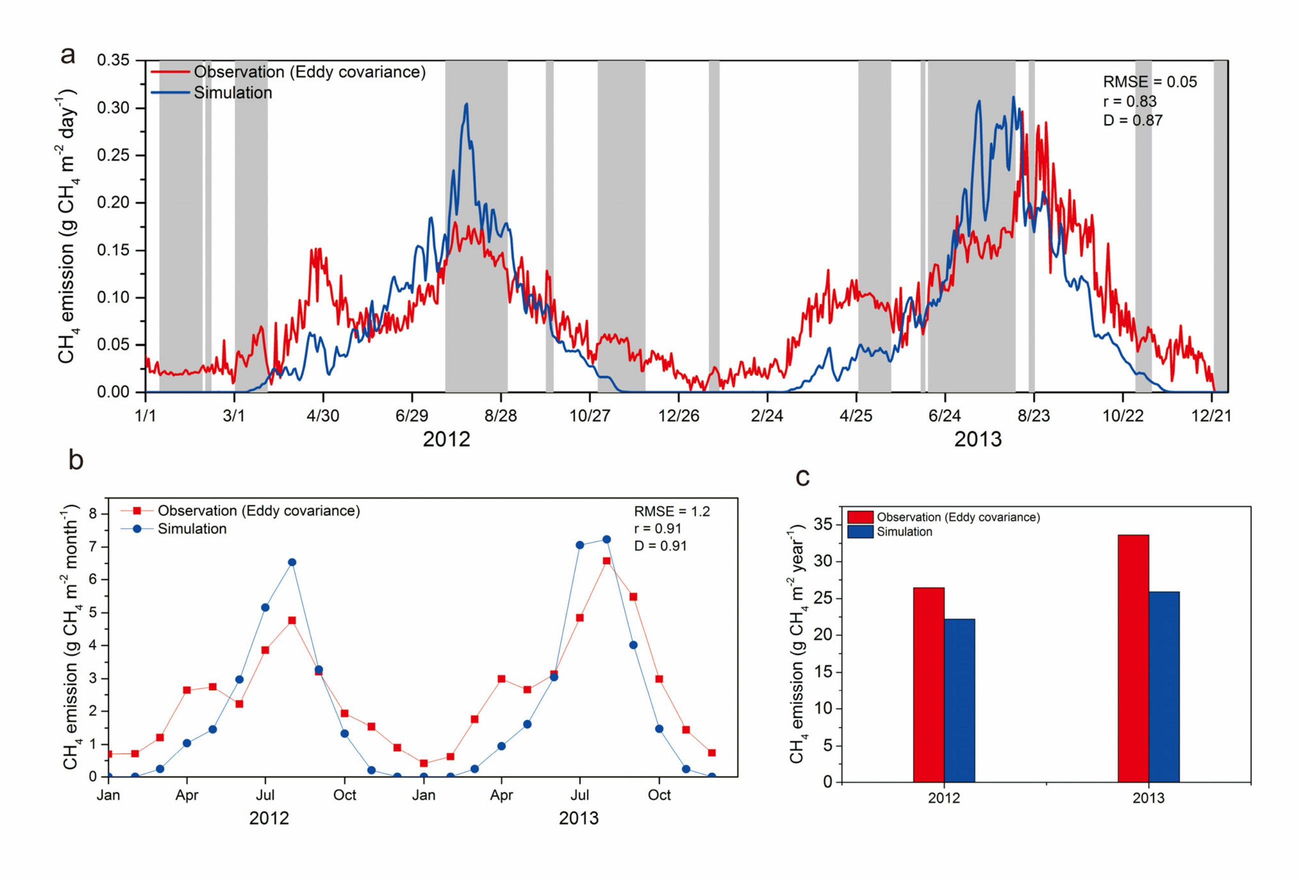

Supplementary Figure S3. Comparison of daily (a), monthly (b), and yearly (c) wetland CH_4 emission of Haibei site with eddy covariance measurement. a. Gray bar in represented the gaps of eddy covariance measurement

2.2 National level estimation and comparison

Early estimations of wetland CH_4 emissions in China, i.e., 1.70 $TgCH_4$ yr^{-1} [8] and 2.20 $TgCH_4$ yr^{-1} [9] around 1990, were conducted using very simple approaches. Based on the observed emission rate at only one permafrost station located in the QTP, Jin et al.[10] extrapolated China's wetland CH_4 emissions at approximately 2.0 $TgCH_4$ yr^{-1} during 1996 and 1997. Using additional data measured in different regions and wetland types, the total wetland CH_4 emission was estimated as 1.76 $TgCH_4$ yr^{-1} around year 2000[11,12]. Using a process model of DLEM, Xu and Tian[13] estimated wetland CH_4 emissions in China as 1.84 $TgCH_4$ yr^{-1} between 2000 and 2008. An estimation using an inventory analysis generated annual wetland CH_4 emissions in China as 2.69 $TgCH_4$ yr^{-1} during the 2000s[14]. The most recent estimation of annual wetland CH_4 emissions of China was 1.61 $TgCH_4$ yr^{-1} around 2009 based on model simulation[15]. Simulated annual wetland CH_4 emissions for the corresponding periods in this study were compared with the above studies, where the range of the variation was consistent (Supplementary Table S3). Our national-level estimation for 1990 was higher than that of previous studies. During the 2000s, our results were similar to the estimation of Xu and Tian[13] and Li et al.[15] but lower than the estimation of Chen et al.[14]. The mean wetland CH_4 emissions for both published literatures and the current modeling study were similar (Supplementary Table S3).

Although our simulated annual total CH_4 emissions from wetlands in China are similar to those of other studies, further explanations should be provided. Earlier CH_4 emission studies have generally lacked detailed temporal or spatial information on estimations[8,9]. In the study of Jin, et al.[10], a uniform CH_4 emission rate (as measured in the QTP) was assumed for all wetlands in the country, including coastal wetlands. In the studies of Ding, et al.[11] and Ding and Cai[12], the methane emission during the unmeasured period was set to 15-23% of that measured in the growing season to calculate the annual

CH_4 emissions, and the estimated wetland area in the studies was approximately 9.4×10^4 km^2, which is lower than that derived in our study. Using the assumption that CH_4 emissions are proportional to the area of wetlands at a national scale, an updated annual CH_4 emissions estimation of approximately 4.5 TgC was suggested in a follow-up study, based on a new evaluation of wetland area[16]. The CH_4 emissions are much larger than those estimated in all other studies to date.

Although the estimated national CH_4 emissions strongly agreed with those of similar studies, considerable differences exist in regional estimations. For example, Chen, et al.[14] found that wetlands in the QTP emitted the most CH_4 in China, as opposed to the NE as suggested in the present study and other studies[11,13]. Extrapolating the relatively higher CH_4 emissions rates measured in peatland on the eastern edge of the QTP to the entire region to estimate total emissions may result in the above overestimated results in QTP. Our study found that the CH_4 emission rate is much lower in the western and northern parts of the QTP than in the eastern part.

Supplementary Table S3. Comparison of wetland CH_4 emissions estimation at national scale between published literatures and our modeling results

This study	Other studies	Time period	Corresponding time in this study	Reference
$TgCH_4$/yr				
2.47	1.70	1990	Year 1990	Khalil et al., 1993
2.47	2.20	1988	Year 1990	Wang et al., 1993
2.03	2.00	1996-1997	Mean of year 1990 and 2000	Jin et al., 1999
2.03	1.76	1995-2004	Mean of year 1990 and 2000	Ding and Cai, 2007
1.59	1.76	2001-2002	Year 2000	Ding et al., 2004
1.75	1.84	2000-2008	Mean of year 2000 and 2008	Xu and Tian, 2012
1.75	2.69	2000s	Mean of year 2000 and 2008	Chen et al., 2013
1.77	1.61	2008-2010	Mean of year 2008 to 2010	Li et al., 2015
1.98	1.95			Mean

Furthermore, most estimations are based on instantaneous CH_4 emission rates measured during

the daytime, and the measurements were only made every two weeks or every month during the growing season. No diurnal variations were considered in the estimation of daily or annual CH_4 emissions. However, CH_4 released from wetlands could have notable diurnal variations, increasing in the morning, reaching a peak at noon, decreasing in the afternoon, and decreasing by approximately 50% to 80% under dark conditions at night[17]. Therefore, bias will be introduced in the estimation of annual CH_4 emissions using the inventory method if diurnal, daily, and seasonal variations are not considered.

3. Relationship between climate (precipitation and temperature) and wetland CH_4 emissions

A long-term analysis (1951-2013) was conducted on the fixed wetlands (i.e., the areas that remained wetlands from 1978 to 2008) to investigate the effects of precipitation and temperature on wetland CH_4 emissions. The correlation coefficient and the significance level between the CH_4 emission rate and precipitation or temperature were calculated for each wetland grid cell, and only those grid cells with statistical significance and a determination coefficient greater than 0.3 are shown in Figure 3b, 3c.

For some northern areas, precipitation was significantly ($P<0.05$) and positively correlated with wetland CH_4 emissions and was responsible for more than 30% of the variation in wetland CH_4 emissions ($R2\geqslant0.3$) (Fig. 3b). Over most wetland areas in the NE, QTP, and SCN regions, non-significant relationships were detected between precipitation and CH_4 emissions.

However, wetland CH_4 emissions were extremely and significantly ($P<0.001$) correlated to temperature in most areas, with an R^2 value greater than 0.3, particularly in the QTP and NE regions (Fig. 3c). For wetlands in the western part of the QTP, the temperature explained more than 60% of the CH_4 emission variation. Combined positive effects of temperature and precipitation were detected in some areas in the northeast QTP and in the northwesternmost region of China (Fig. 3b, 3c).

The high spatial heterogeneity in wetland CH_4 emissions was caused by the substantial spatial variations in the driving variables, including precipitation, temperature, soil temperature, soil moisture, soil text, soil pH and soil redox potential, which control CH_4 production and consumption. The wetlands in South China showed the highest CH_4 emission rates for two reasons. First, abundant precipitation would keep the water table at a relatively high level and maintain good aerobic conditions for methanogenesis. Second, high plant primary production and organic matter decomposition rates provide full substrate availability, which is also an important control over methane production[18].

According to the highly significant positive correlation between CH_4 emissions and temperature outlined in this study, temperature could be one of the most important controls over CH_4 emissions in the high-latitude (NE) and high-altitude (QTP) areas. This link could partially explain why wetlands in the NE and QTP were more sensitive to climate warming than other regions.

In northern China, the significant positive correlation between CH_4 emissions and precipitation indicated that precipitation had a considerable influence on wetland CH_4 emissions. Precipitation

became the limiting factor for the wetlands located in the driest area of China. In the water-limited areas, increased precipitation would increase water table position, reduce the oxic portion of the soil and decrease the oxidative loss of CH_4 [19]. In other regions, temperature is more important to wetland CH_4 emissions than precipitation. For example, the CH_4 emissions are significantly correlated with temperature but not precipitation in the Sanjiang Plains (northeastern China). In a study conducted in this area, Song, et al. [20] also found that CH_4 exponentially increased with temperature but found no significant relationship with water depth (controlled by precipitation). Precipitation has been suggested to have a smaller influence on wetland CH_4 emissions than temperature in China[13] and globally[19]. The bacteria that produce CH_4 has been found to be more sensitive to temperature than other variables and thus a change in temperature may significantly control the emissions[19,21,22].

Only the straightforward relationships between wetland CH_4 emissions and precipitation or temperature are analyzed in this study. CH_4 emissions from wetlands are also influenced by other factors, such as soil redox potential, pH, salinity, the quantity and quality of methanogen substrates, water depth, and topography[18,23-26]. Further investigations should consider these factors, particularly in the areas where CH_4 emissions had no significant relationship with either precipitation or temperature, for example, in the most northern part of Northeast China.

4. Uncertainties and sensitivity analyses of the impacts of wetland dynamics on CH_4 emissions

The remote sensing data based wetland dynamics of China from 1978 to 2008 in this study was derived

from the research of Niu, et al.[27]. The mapping was verified and validated by manually interpretation, with help of other reference data such as digital elevation model (DEM) data, land use/cover data, and Google Earth information[27]. However, some uncertainties still exist. On one hand, it is a challenge to gather all remote sensing images covering the whole country at a specified time, particularly for early years and need to span the time window approximately 3－5 years to complete map over whole country[27]. The wetland dynamic patterns were actually described for a period (around a base year) other than a specific year[27]. On the other hand, the retrieved wetland dynamics based on remote sensing data used in this study could describe a general trend on the patterns of wetland distribution, but unfortunately, further intra-annual dynamics information could not be obtained[27]. Using multiple sources of remote sensing data (e.g. from active and passive sensors) and applying a correlation analysis between wetland change and environmental factors will certainly reduce the uncertainty and improve the accuracy of wetland mapping.

To investigate the impacts of the inter- and intra- annual dynamics of wetland area on wetland CH_4 emissions, additional simulations were carried out using the most recent inundated area dataset of Surface WAter Microwave Product Series (SWAMPS, http://wetlands.jpl.nasa.gov) [28]. From the global dataset, the monthly wetland fraction for each 0.5°×0.5° grid was extracted for the whole China during 2000 to 2012. This additional simulation with wetland distribution data of SWAMPS was split into three periods: the spin-up period, 1951 to 1999, and 2000 to 2012 (Supplementary Table S4). Based on the monthly wetland distribution data of SWAMPS from 2000 to 2012, we generated a multi-year monthly maximum wetland extension dataset, including 12 wetland distribution maps from January to December. For example, the fraction of wetland in a grid cell on the map of January was assigned as

the maximum wetland fraction of January between 2000 and 2012 in the grid cell. During spin-up period, the model was forced with multi-year averaged climate data and monthly maximum wetland extension of SWAMPS. Between 1951 and 1999, the model was forced with daily observed climate data and monthly maximum wetland extension of SWAMPS. Between 2000 and 2012, the model was forced with daily observed climate data and monthly wetland distribution data of SWAMPS (Supplementary Table S4). Monthly total CH_4 emission from wetlands of China between 2000 and 2012 was then used for analysis. The results showed remarkable consistency in seasonal cycle patterns between wetland CH_4 emissions and wetland area dynamics (Supplementary Fig. S4a), and wetland area changes could explain the variations of wetland CH_4 emissions at approximately 86% (Supplementary Fig. S4b). Seasonal and interannual wetland area variations could be the major uncertainties in the wetland CH_4 emissions. Wetland distribution is another one of the important factors that affect total wetland CH_4 emissions as well as the magnitude of CH_4 emission rates. Here, the wetland distribution derived from SWAMPS dataset is quite coarse which could omit a considerable amount of small wetland patches in China. Recently, remote sensed data have been used to derive monthly global inundated areas[28-30] and make it possible to quantify the impact of wetland extent changes on CH_4 emissions at the seasonal and interannual time scales. However, it is still difficult to acquire historic wetland distributions and use them for long-term simulation experiments due to lacking of remote sensing data. Land surface models integrated with detailed hydrological processes could potentially provide an effective method to estimate wetland area dynamics[5].

Supplementary Table S4. Simulations conducted with monthly wetland distribution data of SWAMPS

Simulation forcing data	Spin-up (300yr)	1951-1999	2000-2012
Climate	Multi-year (1960-2000) averaged	Daily observed data	
Wetland distribution	Monthly maximum wetland extension of SWAMPS		Monthly wetland distribution of SWAMPS

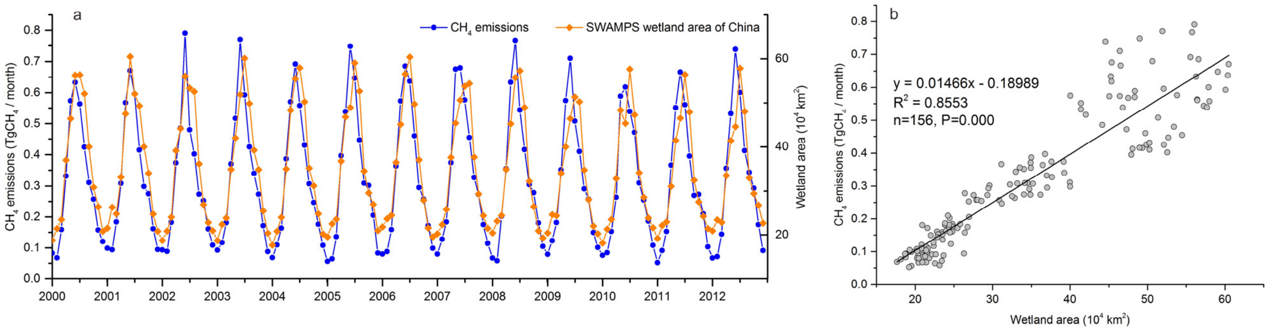

Supplementary Figure S4. Correlation between monthly wetland CH_4 emissions and monthly wetland area changing in China based on SWAMPS during 2000 and 2012: (a) Temporal patterns comparison, (b) Scatter comparison and linear regression.

5. Wetlands mapping of China based on remotely sensed data

The wetland area dynamics, one of the input data for the model, was directly derived from the previous works of Niu et al. which were conducted between 2008 and 2012 [27,31-35]. Here we presented a brief description of wetlands mapping of China based on remotely sensed data and more details could be found in the references [27,31-35].

Different sources of satellite imagery were used to produce the national wetland maps for years 1978, 1990, 2000, and 2008 maps: MSS, Landsat TM, ETM+, and CBERS-02B CCD, respectively [27,33]. Atmospheric correction of images was undertaken based on the Fast Line-of-sight Atmospheric Analysis of Spectral Hypercubes (FLAASH) method with ENVI (The Environment for Visualizing Images) software (Exelis Visual Information Solutions, Inc.)[27,33]. Landsat TM images around 1990 served as reference images, and the other period images (1978, 2000, and 2008) were corrected by

using the image-to-image method with ENVI software. The georegistration error was constrained to within two pixels for CBERS-02B images and less than one pixel for ETM and MSS as measured by root-mean-square error[27,33]. Given the heterogeneity of China's landscape, manual interpretation was chosen over automation to map wetland vegetation and most of the classification was done by visual interpretation[27,33,34].

Accuracy assessment was also made for the wetland mapping. For example, in the study of Gong et al.[32], the uncertainty and interpretation errors of wetland area change between 1990 and 2000 were evaluated from two aspects: misregistration and misinterpretation. The conversion error matrix indicated that inland natural wetlands has the false alarm error between 1% and 2% [32]. Weighted by area, the total error in change area statistics due to misregistration-caused false alarm would be less than 2% [32]. The overall image interpretation errors for inland natural wetlands were estimated to be 1.3% [32]. By taking into account the misinterpretation of wetland classes, the overall error for the inland natural wetlands should be less than 5% [32].

Wetland reconnaissance for the 2008 map was undertaken from July 2009 to September 2009 and more than 10,000 field photos were taken and 459 qualified samples (located at representative wetland regions including the Qinghai-Tibet Plateau, the Northeast Plain, the Yellow River delta, east and southeast of China) were collected to validate the quality of wetland mapping for the period of 2008 [27]. The overall accuracy of wetland and nonwetland was 0.98 while the overall accuracy of wetland types was 0.70 and kappa coefficient is 0.63[27,33] . Both of producer accuracy and user accuracy for inland wetlands were 0.83[33]. After validation of the wetland map in 2008, the other three maps (circa

2000, 1990, and 1978) were intersected, and a cross-table of change between every two-period wetland type was constructed [33]. According to the change model of wetland types between different periods, combinations were identified as impossible wetland transformation, such as from coastal wetlands to inland wetlands, and were rechecked on the images, and the corresponding map was revised accordingly [27,33]. Auxiliary materials and literature related to wetland distribution were collected to validate regions we could not confirm during the visual interpretation. The Google Earth information was fully utilized for the visual interpretation and validation processes [27,33].

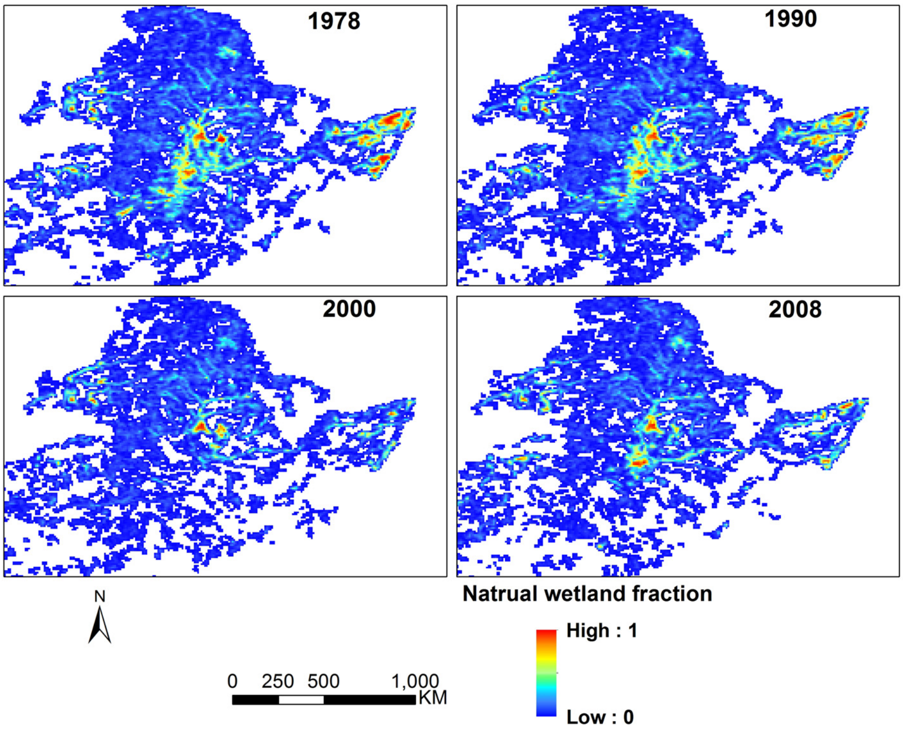

Supplementary Figure S5. An example of natural wetland fraction distribution of Northeast China. The maps were generated with ArcGIS 10.2, http://www.esri.com/.

Acknowledgement: We would like to thank Ben Poulter, from Montana State University, for providing a global bias-corrected wetland fraction dataset which was originated from the SWAMPS dataset.

References:

1 Keeling, C. D. *et al.* in *A History of Atmospheric CO_2 and its effects on Plants Animals and Ecosystems* (eds J.R. Ehleringer, T. E. Cerling, & M. D. Dearing) 83-113 (Springer Verlag, 2005).

2 Enting, I., Heimann, M. & Wigley, T. Future emissions and concentrations of carbon dioxide: Key ocean/atmosphere/land analyses. (CSIRO Division of Atmospheric Research Technical Paper No. 31, CSIRO, Australia, 1994).

3 Zhu, Q. *et al.* Modelling methane emissions from natural wetlands by development and application of the TRIPLEX-GHG model. *Geosci Model Dev* **7**, 981-999 (2014).

4 Wania, R., Ross, I. & Prentice, I. C. Integrating peatlands and permafrost into a dynamic global vegetation model: 2. Evaluation and sensitivity of vegetation and carbon cycle processes. *Global Biogeochem Cy* **23**, GB3015, doi:10.1029/2008gb003413 (2009).

5 Zhu, Q. *et al.* Estimating global natural wetland methane emissions using process modelling: spatio-temporal patterns and contributions to atmospheric methane fluctuations. *Global Ecology and Biogeography* **24**, 959-972 (2015).

6 Li, T. T., Huang, Y., Zhang, W. & Song, C. C. CH4MOD(wetland): A biogeophysical model for simulating methane emissions from natural wetlands. *Ecological Modelling* **221**, 666-680, doi:DOI 10.1016/j.ecolmodel.2009.05.017 (2010).

7 Song, W. *et al.* Methane emissions from an alpine wetland on the Tibetan Plateau: Neglected but vital contribution of the nongrowing season. *Journal of Geophysical Research: Biogeosciences*, 10.1002/2015JG003043, doi:10.1002/2015JG003043 (2015).

8 Khalil, M. A. K., Shearer, M. J. & Rasmussen, R. A. Methane Sources in China - Historical and Current Emissions. *Chemosphere* **26**, 127-142 (1993).

9 Wang, M., Dai, A., Huang, J., Ren, L. & Shen, R. Estimate of methane emission from china. *Chinese Journal of Atmospheric Sciences* **17**, 49–62 (1993).

10 Jin, H., Wu, J., Cheng, G., Nakano, T. & Sun, G. Methane emissions from wetlands on the Qinghai-Tibet Plateau. *Chinese Science Bulletin* **44**, 2282-2286, doi:10.1007/bf02885940 (1999).

11 Ding, W., Cai, Z. & Wang, D. Preliminary budget of methane emissions from natural wetlands in China. *Atmospheric Environment* **38**, 751-759, doi:10.1016/j.atmosenv.2003.10.016 (2004).

12 Ding, W. X. & Cai, Z. C. Methane emission from natural wetlands in China: Summary of years 1995-2004 studies. *Pedosphere* **17**, 475-486, doi:Doi 10.1016/S1002-0160(07)60057-5 (2007).

13 Xu, X. F. & Tian, H. Q. Methane exchange between marshland and the atmosphere over China during 1949-2008. *Global Biogeochem Cy* **26**, GB2006, doi:2010.1029/2010GB003946 (2012).

14 Chen, H. *et al.* Methane emissions from rice paddies natural wetlands, lakes in China: synthesis new estimate. *Global Change Biology* **19**, 19-32 (2013).

15 Li, T. *et al.* Impacts of climate and reclamation on temporal variations in CH4 emissions from different wetlands in China: from 1950 to 2010. *Biogeosciences Discuss.* **12**, 7055-7091, doi:10.5194/bgd-12-7055-2015 (2015).

16 Cai, Z. C. Greenhouse gas budget for terrestrial ecosystems in China. *Sci China Earth Sci* **55**, 173-182 (2012).

17 Hirota, M. *et al.* Methane emissions from different vegetation zones in a Qinghai-Tibetan

Plateau wetland. *Soil Biol Biochem* **36**, 737-748, doi:DOI 10.1016/j.soilbio.2003.12.009 (2004).
18 Cao, M. K., Marshall, S. & Gregson, K. Global carbon exchange and methane emissions from natural wetlands: Application of a process-based model. *J Geophys Res-Atmos* **101**, 14399-14414, doi:10.1029/96jd00219 (1996).
19 Melton, J. R. *et al.* Present state of global wetland extent and wetland methane modelling: conclusions from a model inter-comparison project (WETCHIMP). *Biogeosciences* **10**, 753-788, doi:DOI 10.5194/bg-10-753-2013 (2013).
20 Song, C., Xu, X., Tian, H. & Wang, Y. Ecosystem-atmosphere exchange of CH_4 and N_2O and ecosystem respiration in wetlands in the Sanjiang Plain, Northeastern China. *Global Change Biology* **15**, 692-705 (2009).
21 EPA. Methane and Nitrous Oxide Emissions From Natural Sources (EPA 430-R-10-001). (2010).
22 Turetsky, M. R. *et al.* A synthesis of methane emissions from 71 northern, temperate, and subtropical wetlands. *Global Change Biology* **20**, 2183-2197, doi:Doi 10.1111/Gcb.12580 (2014).
23 Le Mer, J. & Roger, P. Production, oxidation, emission and consumption of methane by soils: A review. *Eur J Soil Biol* **37**, 25-50, doi:http://dx.doi.org/10.1016/S1164-5563(01)01067-6 (2001).
24 Moore, T. R. & Knowles, R. Methane Emissions from Fen, Bog and Swamp Peatlands in Quebec. *Biogeochemistry* **11**, 45-61 (1990).
25 Segers, R. Methane Production and Methane Consumption: A Review of Processes Underlying Wetland Methane Fluxes. *Biogeochemistry* **41**, 23-51 (1998).
26 Waddington, J. M., Roulet, N. T. & Swanson, R. V. Water table control of CH4 emission enhancement by vascular plants in boreal peatlands. *J Geophys Res-Atmos* **101**, 22775-22785 (1996).
27 Niu, Z. *et al.* Mapping wetland changes in China between 1978 and 2008. *Chinese Science Bulletin* **57**, 2813-2823 (2012).
28 Schroeder, R. *et al.* Development and Evaluation of a Multi-year Inundated Land Surface Data Set Derived from Active/Passive Microwave Remote Sensing Data: Assembly of a Global-scale Inundated Wetlands Earth System Data Record. *In preparation* (2014).
29 Papa, F. *et al.* Interannual variability of surface water extent at the global scale, 1993-2004. *Journal of Geophysical Research D: Atmospheres* **115**, D12111,doi:12110.11029/12009JD012674 (2010).
30 Prigent, C., Papa, F., Aires, F., Rossow, W. B. & Matthews, E. Global inundation dynamics inferred from multiple satellite observations, 1993-2000. *Journal of Geophysical Research D: Atmospheres* **112**, D12107, doi:12110.11029/12006JD007847 (2007).
31 Cyranoski, D. Putting China's wetlands on the map. *Nature* **458**, 134-134 (2009).
32 Gong, P. *et al.* China's wetland change (1990-2000) determined by remote sensing. *Sci China Earth Sci* **53**, 1036-1042 (2010).
33 Niu, Z. in *Remote Sensing of Wetlands: Applications and Advances* (eds Ralph W. Tiner, Megan W. Lang, & Victor V. Klemas) Ch. 22, 473-490 (CRC Press, 2015).
34 Niu, Z. *et al.* Geographical characteristics of China's wetlands derived from remotely sensed data. *Sci China Earth Sci* **52**, 723-738 (2009).
35 Niu, Z. G., Zhang, H. Y. & Gong, P. More protection for China's wetlands. *Nature* **471**, 305-305 (2011).
